# Reference intervals and values for fecal cortisol, aldosterone, and the ratio of cortisol to dehydroepiandrosterone metabolites in four species of cetaceans

**DOI:** 10.1371/journal.pone.0250331

**Published:** 2021-08-30

**Authors:** Lance J. Miller, Lisa K. Lauderdale, Michael T. Walsh, Jocelyn L. Bryant, Kevin A. Mitchell, Douglas A. Granger, Jill D. Mellen

**Affiliations:** 1 Conservation Science and Animal Welfare Research, Chicago Zoological Society–Brookfield Zoo, Brookfield, Illinois, United States of America; 2 Department of Comparative, Diagnostic & Population Medicine, College of Veterinary Medicine, University of Florida, Gainesville, FL, United States of America; 3 Institute for Interdisciplinary Salivary Bioscience Research, University of California, Irvine, CA, United States of America; 4 Biology Department, Portland State University, Portland, OR, United States of America; Universita di Bologna, ITALY

## Abstract

The goal of the current study was to create reference intervals and values for several common and one potential novel physiological indicators of animal welfare for four species of cetaceans. The subjects included 189 bottlenose dolphins (*Tursiops truncatus*), 27 Indo-Pacific bottlenose dolphins (*Tursiops aduncus*), eight Pacific white-sided dolphins (*Lagenorhynchus obliquidens*), and 13 beluga whales (*Delphinapterus leucas*) at Alliance of Marine Mammal Parks and Aquariums and/or Association of Zoos and Aquariums accredited facilities. During two sampling time periods between July and November of 2018 and between January and April of 2019, fecal samples were collected weekly for five weeks from all animals. Samples were processed and analyzed using enzyme immunoassay for fecal cortisol, aldosterone, and dehydroepiandrosterone (DHEA) metabolites. Linear mixed models were used to examine demographic and time factors impacting hormone metabolite concentrations. Age, sex, and time of year were all significant predictors for some of the models (p < 0.01). An iOS mobile application ZooPhysioTrak was created for easy access to species-specific reference intervals and values accounting for significant predictors. For facilities without access to this application, additional reference intervals and values were constructed without accounting for significant predictors. Information gained from this study and the use of the application can provide reference intervals and values to make informed management decisions for cetaceans in zoological facilities.

## Introduction

Accredited zoological facilities regularly monitor the health and welfare of the animals under their professional care through a variety of ways including regularly scheduled proactive health exams. These exams are a requirement of both the Alliance of Marine Mammal Parks and Aquariums (AMMPA) and the Association of Zoos and Aquariums (AZA). Less-invasive tracking of adrenal hormone levels through fecal samples is one method of monitoring the welfare of animals [1, 2]. In the case of cetaceans, animals can be trained to voluntarily provide fecal samples collected through the use of a sterile catheter as part of their routine preventive health program [3]. This approach to fecal collection in an aquatic species has provided a method to track an individual’s hormone levels over time using enzyme-immunoassays. This information can help animal care staff make informed management decisions based on how animals are responding to changes within their environment to ensure the animals are thriving. Examples of changes that could be monitored include changes in social groups, transport between facilities, handling techniques, or changes in daily routines. Three potentially useful hormones as markers of adrenal activity include cortisol, aldosterone, and dehydroepiandrosterone (DHEA).

Cortisol, a glucocorticoid produced by the adrenal gland, increases in production under times of stress, exercise, and arousal [4]. Glucocorticoids are responsible for maintaining homeostasis through the increased secretion of glucose resulting in the needed energy to address an environmental change, while also inhibiting non-essential functions [4, 5]. Cortisol is the main glucocorticoid produced by most mammals [6] and has been studied in a variety of cetaceans [1, 3, 7]. Historically, the majority of the studies examining cortisol in cetaceans has been done using blood samples [7]. However, the act of collecting those samples could have an impact on the values as has been seen in other species [8–10] and blood samples represent a very short period of time. Fecal samples on the other hand can be collected less-invasively, and represent a longer period of time so they may be more suited for monitoring animal welfare [11]. Under professional care in zoos and aquariums, animals are often already trained or can be trained to provide fecal samples. Voluntary collection of fecal samples can be much less invasive and provide multiple samples to track animals over time [3]. While non-repetitive acute stressors typically do not negatively impact welfare, chronic activation of the hypothalamic-pituitary- adrenal (HPA) axis can be associated with and result in reduction of an animal’s welfare state [12, 13].

While cortisol has been studied across a variety of cetaceans, aldosterone has been suggested as another marker of adrenal function that when analyzed alongside cortisol, may provide a more complete picture of adrenal activity [14]. Aldosterone, a mineralocorticoid produced by the adrenal gland, is also part of the HPA axis response [15]. Aldosterone has many different functions including electrolyte balance and water retention in the kidneys. During a stress response, aldosterone appears to play a role in stabilizing blood pressure [15]. Aldosterone has primarily been studied in smaller cetaceans [7, 16]; however there have been studies on larger animals [14]. However, there have been some challenges around circulating levels for bottlenose dolphins falling under the detection levels of commercial assay kits [16]. More research is needed to better understand the importance of aldosterone in monitoring adrenal activity in cetaceans.

DHEA is known to impact knowledge retention, have anti-aging properties, enhance the immune system, and mental health in humans [5, 17, 18]. DHEA and its sulfate ester (DHEA-S) are the most abundant hormones produced for some species [19]. DHEA and DHEA-S have been studied in a variety of species ranging from rabbits to horses but have only been examined in a few studies with cetaceans [20–23]. However, examination of the human literature suggests that DHEA by itself might not be the most useful measure of welfare, but may be more meaningful when considered as a ratio in relation to cortisol levels. The ratio of cortisol to DHEA has been a significant predictor of mental health in humans [24, 25] and is likely a much better indicator of how the HPA axis is functioning then looking at either hormone by itself [4]. When a person is chronically stressed or experiences repeated acute stressors, this may impact the balance between cortisol and DHEA leading to effects on physical, mental, and emotional health [5]. While additional information is clearly needed for cetaceans, the ratio of cortisol to DHEA might be a tool to help better understand how individual animals are faring at any given point in time or over an extended period.

While previous research has created cortisol and aldosterone reference intervals for bottlenose dolphins using blood samples [16], to the best of our knowledge there have been no studies to create reference intervals or values utilizing less invasive fecal samples. The goal of the current study was to create reference intervals and values for fecal cortisol, aldosterone, and cortisol:DHEA metabolites in four species of cetaceans. These include common bottlenose dolphins (*Tursiops truncatus*), Indo-Pacific bottlenose dolphins (*Tursiops aduncus*), Pacific white-sided dolphins (*Lagenorhynchus obliquidens*) and beluga whales (*Delphinapterus leucas*). Reference intervals are ranges of values from healthy individuals used by veterinarians to evaluate the health of an individual animal that are developed from a dataset with a large sample size. Reference values are ranges of values for animals that are developed from a dataset with a small sample size. With reference intervals and values, facilities can track hormone levels to determine if an animal falls within a normal range. Tracking these hormones can also serve as an animal management tool to determine how management, illness, or environmental changes impact animal welfare.

## Methods

### Ethics statement

The current study was approved by animal care and veterinary staff at each institution. In addition, the U.S. Navy Marine Mammal Program Institutional Animal Care and Use Committee reviewed and approved the project #123–2017.

### Subjects and facilities

The subjects included 189 common bottlenose dolphins, 27 Indo-Pacific bottlenose dolphins, eight Pacific white-sided dolphins, and 13 beluga whales. Given the sample size, the ranges created for common bottlenose dolphins would be considered reference intervals while the ranges for the other three species would be considered reference values. Samples from common bottlenose dolphins came from 41 different facilities, samples from Indo-Pacific bottlenose dolphins came from two facilities, samples from Pacific white-sided dolphins came from four facilities, and samples from beluga whales came from two facilities. In total, the subjects were located at 43 unique zoological facilities that were accredited by the AMMPA and/or AZA. This current research is part of the larger effort entitled “Towards Understanding the Welfare of Cetaceans in Zoos and Aquariums.”

### Data collection

Two separate five-week data collection periods separated by approximately six months were used to have a range of values from two different seasons. The first five-week period of data collection occurred between July and November of 2018 and the second five-week period of data collection occurred between January and April of 2019. Each facility collected one fecal sample per focal animal each week over each five-week period for a maximum of ten samples per animal. Fecal samples were collected during the last two hours of the day when staff were present at each facility usually between the hours of 16:00 and 18:00 local time. Collecting at this time accounted for any diurnal patterns in hormone metabolite levels and also allowed for collection of more fecal material due to animals feeding throughout the day. A clean disinfected catheter was used to collect the samples as animals were trained to voluntarily participate in sample collection and ensure there was no saltwater contamination of the sample. The catheters were 40.64 cm long, flexible, size 15 French for the three larger species, and size 10 French for the Pacific white-sided dolphins. In order to prevent contamination due to exogenous hormones, staff wore disposable gloves during sample collection. Samples were collected from the animals while positioned ventral side up and parallel to the animal care staff’s platform. Fecal material was allowed to fill passively via capillary action and then pinched at the top to prevent material from exiting the catheter will being gently removed from the animals. If less than 2 mL of material were collected, staff discarded the sample and tried to collect the following day. Sterile water was run through the catheter if samples were too viscous and transferred directly to a pre-labeled 5ml vial. Vials with the samples were immediately transferred to a freezer and stored frozen at -20°C. At the end of data collection, samples from all animals at a facility were shipped on dry ice to the Chicago Zoological Society Endocrine Laboratory.

Fecal samples were freeze dried in a lyophilizer (Labconco model #A65412906 with Edwards oil mist filter model #EMF10) for 48 hours at -50°C at 0.06 mBar. After drying, samples were manually ground and 0.025 g ± 0.005 g of homogenized sample were weighed and transferred to a 2.5mL polypropylene screw capped tube until further analysis. In order to avoid the effects of small samples on hormone concentrations, fecal samples with less than 0.02 g remaining were excluded from further analysis [26, 27].

Hormone analysis was based on small modifications to previously established protocols [28, 29]. In brief, fecal hormone metabolites were extracted using 500 uL 80% ethanol in dH2O, yielding a ratio of 2.5ml of 80% ethanol per 0.1g of dried sample. However, extraction efficiency was not measured and could be a source of variability. Samples were then placed overnight on a rotator (Labline Maxi Rotator catalog no. 4631) and centrifuged (Fisher Marathon 3000R) at 2500 rpm for 20 minutes. 200 uL of supernatant was transferred into a new 1.5mL microcentrifuge tube. Commercially available enzyme immunoassay (EIA) kits from Arbor Assays (cortisol, catalog no. K003-H5; aldosterone, catalog no. KO52-H5) and Genway BioTech (DHEA, catalog no. GWB-719A7E) were used for analysis. Fecal hormone metabolites were quantified using each corresponding manufactures’ instructions. All samples were run in duplicate and plates were read using a Dynex Technologies MRX Microplate Reader with Revelation software at 405nm. Any samples with greater than 15% CV between duplicates were reassayed.

All assay validation steps were taken utilizing samples from *Tursiops truncatus*. Assay validation steps were not performed for *Tursiops aduncus*, *Lagenorhynchus obliquidens*, and *Delphinapterus leucas* given their taxonomic relatedness and previous research. Cortisol, aldosterone, and DHEA assay kits have all been used and validated with a variety of cetaceans utilizing both blood and fecal samples depending on the analyte [3, 5, 7–9, 16, 20, 30]. Biochemical validation consisted of examining parallelism with the standard curve. Serial two-fold dilutions of a sample pool with mixed sex and age were tested for parallel displacement curves on each EIA. In addition, the percentage of exogenous hormone was measured using a recovery test. Recovery of exogenous hormone was measured by adding a known amount of hormone to diluted samples. The recovery was measured across six concentrations. Variability between assays was monitored using high and low controls and intra-assay variability was measured by adding the same sample to several wells within one plate.

Cross reactivities in the cortisol EIA were: 100% cortisol, 18.8% dexamethasone, 7.8% prednisolone, 1.2% corticosterone, 1.2% cortisone, and all other cross reactants were below 0.1%. Serial dilutions for the cortisol assay demonstrated good parallelism (F(1,5) = 55.379, p < 0.001). Assay sensitivity was determined to be 27.6 pg/mL (standard range: 50 pg/mL-3200 pg/mL) and a 96.7% mean recovery of exogenous cortisol. Inter-assay variation was calculated using a high and low control that yielded a result of 9.97% CV at 44.34% binding and 8.04% at 23.72% binding. Intra-assay variation was 9.03%.

For the aldosterone EIA, cross reactivities were as follows: 100% aldosterone, 0.047% corticosterone, 0.019% desoxycorticosterone, and all other reactants were less than 0.016%. Serial dilutions for the aldosterone assay did not demonstrate as good of parallelism as the cortisol assay (F(1,5) = 8.660, p = 0.060). Assay sensitivity was 4.97 pg/mL (standard range: 3.906 pg/mL-4000 pg/mL) with a mean recovery of 100.7% exogenous hormone. Inter-assay coefficients of variation were 15.66% at 89.54% binding and 20.75% at 12.99% binding. The intra-assay coefficient of variation was 11.58%.

The cross reactivities for the DHEA EIA were as follows: 100% DHEA, 4.555% epiandrosterone, 0.610% androstenedione, 0.496% testosterone, 0.471% DHT, 0.419% progesterone, and all other compounds were less than 0.3%. Serial dilutions for the DHEA assay also demonstrated good parallelism (F(1,5) = 33.633, p < 0.05). The sensitivity of this assay is 0.082 ng/mL (standard range: 0.2 ng/mL-40 ng/mL) with a mean recovery of 101.89% exogenous hormone. The inter-assay coefficients of variation were calculated to be 7.60% at 21.10% binding and 9.12% at 9.49% binding. The intra-assay coefficient of variation was 0.384%. All fecal hormone concentrations were calculated as ng/g dry weight for use in statistical analysis.

### Statistical analysis

For the creation of reference intervals and values for physiological data from fecal samples it was critical to ensure that the animals were healthy at the time of collection. Only samples from known healthy whales and dolphins were included in the final analysis. A whale or dolphin was considered healthy if had no known disease at the time of the study, and was not taking medications that could impact the physiological values of interest. Animals were determined to be healthy based on a blinded full review of health survey and bloodwork by an expert veterinarian as well as blinded review of cytology results from chuff, fecal, and gastric samples by a clinical pathologist. While we took many steps to ensure the animals in the current study were healthy through the veterinary exams and review of health data, we cannot guarantee that the animals in the final analysis were not experiencing some level of stress, and that variability is likely captured in the range of values. Animals that were taking eye drops, nutritional supplements, or contraceptives were included in the final analysis.

Statistical analyses were conducted using SPSS 21 and Microsoft Excel software packages. Mixed models were used to compare fecal hormone values by sex, age, age^2^, and month (controlling for facility ID and animal ID) for common bottlenose dolphins, Indo-Pacific bottlenose dolphins, and beluga whales. Significance was defined as p < 0.01. Seventy percent of the data were separated as a training data set and the other 30% were separated into a testing set. Observations were randomly separated by individual in order to ensure a variety of locations, sample months, sex classes, and ages were included in each set. Full mixed models were run with sex, age, age^2^, or month variables. Significant predictors were selected, and mixed models were developed with only those parameters. For example, if sex and age were significant predictors in the model, then a model with only sex and age was created and those parameters were included in the reference interval or value equations.

To allow prediction equations to be useful with future samples, we excluded animal ID and facility ID and produced reduced linear models. Consistent with the mixed model protocol, significant differences were identified in models that included sex, age, age^2^, and month variables. Models were then developed with only significant variables and reference intervals or values were developed using the associated significant parameters. If no significant predictors were identified in the model, reference intervals or values were calculated from values within two standard deviations from the mean. The prediction equations generated from the training dataset models were solved for the testing dataset. The actual values were compared to the resulting predicted values from the testing dataset by assessing the reduced linear model output, means, and standard deviations.

Reference intervals or values (*i*.*e*., 95% confidence intervals) were developed for variables with significant predictors using the following function:

$$95\%\;\mathrm{CI}=\hat{Y}+/‐\left(\left(t_{\mathrm{crit}}\right)\left({\tilde{sd}}_{Y_{o}-\hat{Y_{o}}}\right)\right)$$

where $\hat{Y}$ = predicted value, t_crit_ = critical value, and ${\tilde{sd}}_{Y_{o}-\hat{Y_{o}}}$ = standard error of the estimated Y for a single observation. The standard error of the estimated Y for a single observation with one predictor was calculated as:

$${\tilde{sd}}_{Y_{o}-\hat{Y_{o}}}={\tilde{sd}}_{Y-\hat{Y}}\sqrt{1+\frac{1}{n}+\frac{{\left(X_{o}-\bar{X}\right)}^{2}}{n{sd}_{X}^{2}}}$$

where ${\tilde{sd}}_{Y-\hat{Y}}$ = standard error of the estimate, *n* = sample size, *X_o_* = score on the predictor, $\bar{X}$ = mean of the predictor, *sd_X_* = standard deviation of the predictor [28].

For variables with multiple predictors, the standard error of the estimated Y for a single observation was calculated as:

$${\tilde{sd}}_{Y_{o}-\hat{Y_{o}}}=\frac{{\tilde{sd}}_{Y-\hat{Y}}}{\sqrt{n}}\sqrt{n+1+\sum \frac{z_{io}^{2}}{1-R_{i}^{2}}-2\sum \frac{\beta_{ij}z_{io}z_{jo}}{1-R_{i}^{2}}},$$

where the first summation is over *k* IVs and the second summation is over *k*(*k*-1)/2 pairs of IVs expressed as standard scores, ${\tilde{sd}}_{Y-\hat{Y}}$ = standard error of the estimate, *z_io_* = absolute value of the IV, $R_{i}^{2}=R_{i∙12\ldots (i)\ldots k}^{2}$, and *β_ij_* = standardized partial regression coefficient [31].

Non-parametric reference values were generated for Pacific white-sided dolphins because of their small sample size. To develop the reference values, data were ranked in order of magnitude. The values that fell between the 10th and 90th percentiles were retained as the reference values [29].

### ZooPhysioTrak

In order to use the prediction equations at zoological facilities, an iOS application was created that can be utilized on the iPad, iPhone, and iPod Touch. The application does not require internet connection to operate and is completely customized for this project. The application was programmed using the Swift language and initial versions were tested and distributed using TestFlight. The application was flexible and designed with the ability to be easily updated in the future for additional species or new indicators of animal welfare. When the application was built, the process included automating unit testing to ensure accurate results. This involved several tests including a walkthrough of the statistical analysis using an example dataset and display of the results. Additionally, within the application’s database variables were also tested to ensure calculation for that variable was correct. Finally, additional tests ensured data were internally consistent and values were displayed in the desired order. The application was made freely available to any facilities that care for these four species of cetaceans.

## Results

Prediction equations for the training and testing dataset and r^2^ can be found in Table 1. This includes equations for cortisol, aldosterone, and DHEA fecal metabolites as well the cortisol:DHEA ratio. Mean differences between the observed and predicted values generated from the models are in Table 2. Linear mixed models (Table 3) and reduced linear models (Table 4) highlight the significant demographic and time factors impacting hormone values. Reduced models with significant predictors were used in ZooPhysioTrak. Facilities without the ZooPhyioTrak application can use reference intervals or values provided in Table 5 that do not account for the significant factors presented in Table 4.

**Table 1:** Fit of predictions on training and testing dataset from linear regression models.

Variable	Data Set	n	Equation	r^2^
**Species: *Tursiops truncatus***				
Cortisol (ng/g)	Training	972	164.03 + 1.795 * Age	0.016
	Testing	418	173.921 + 1.593 * Age	0.006
Aldosterone (ng/g)	Training	972	40.708 + 1.394 * Month	0.007
	Testing	418	45.046 + 2.542 * Month	0.005
DHEA (ng/g)	Training	972	1399.99 + -17.076 * Month	0.008
	Testing	418	1379.469 + -3.762 * Month	<0.001
**Species: *Tursiops aduncus***				
Cortisol (ng/g)	Training	162	194.985 + 19.28 * Month	0.065
	Testing	70	205.692 + 16.844 * Month	0.023
Aldosterone (ng/g)	Training	161	133.979 + -11.45 * Month + -22.483 * Sex	0.359
	Testing	70	136.442 + -10.304 * Month + -35.14 * Sex	0.197
DHEA (ng/g)	Training	161	1513.886 + -226.034 * Sex + 12.928 * Age + -38.039 * Month	0.151
	Testing	70	2456.908 + -552.262 * Sex + -6.213 * Age + -91.828 * Month	0.065
Cort:DHEA Ratio	Training	161	.131 + .016 * Month	0.102
	Testing	70	.073 + .025 * Month	0.306
**Species: *Delphinapterus leucas***				
Cort:DHEA Ratio	Training	54	.095 + .003 * Age	0.205
	Testing	22	.079 + .003 * Age	0.238

*Note*. n is the total number of samples.

**Table 2:** Mean difference between observed and predicted values generated from the linear regression models.

Variable	n	Mean Difference	Standard Deviation Difference
**Species: *Tursiops truncatus***			
Cortisol (ng/g)	418	6.48	193.21
Aldosterone (ng/g)	418	47.89	837.55
DHEA (ng/g)	418	10.36	121.75
**Species: *Tursiops aduncus***			
Cortisol (ng/g)	70	-1.72	236.09
Aldosterone (ng/g)	70	132.02	1337.77
DHEA (ng/g)	70	1.26	41.70
Cort:DHEA Ratio	70	-0.01	0.07
**Species: *Delphinapterus leucas***			
Cort:DHEA Ratio	22	-0.03	0.02

*Note*. n is the total number of samples.

**Table 3:** Model estimates for predicting fecal hormone values. Mixed models included the explanatory variables found to be significant predictors. Possible fixed effects included: sex, age, age^2^, and month. Random effects included: animalID and facilityID.

Model	Variable	Parameter	Estimate		Standard Error	*T*	*p*
**Species: *Tursiops truncatus***							
i	Aldosterone (ng/g)	Intercept	40.09		3.69	10.88	< 0.001
		Month	1.50		0.58	2.61	0.009
**Species: *Tursiops aduncus***							
ii	Cortisol (ng/g)	Intercept	229.71		36.00	6.38	< 0.001
		Month	15.16		5.42	2.80	0.006
iii	Aldosterone (ng/g)	Intercept	119.80		8.71	13.75	< 0.001
		Month	-11.02		1.37	-8.02	< 0.001
iv	Cort:DHEA Ratio	Intercept	0.14		0.02	5.90	< 0.001
		Month	0.02		0.004	3.78	< 0.001
**Random Factors**		**Variance**		**Standard Deviation**			
**Species: *Tursiops truncatus***							
AnimalID		1367089.58		1169.23			
FacilityID		194.76		13.96			
**Species: *Tursiops aduncus***							
AnimalID		1348942.00		1161.44			
FacilityID		73.61		8.58			
**Species: *Delphinapterus leucas***							
AnimalID		1268342.38		1126.21			
FacilityID		9.43		3.07			

**Table 4:** Model estimates for predicting fecal hormone values. Linear regression models included the explanatory variables found to be significant predictors. Possible fixed factors included: sex, age, age^2^, and month.

Model	Variable	Parameter	Estimate	Standard Error	*T*	*p*
**Species: *Tursiops truncatus***						
i	Cortisol (ng/g)	Intercept	164.03	9.06	18.11	< 0.001
		Age	1.80	0.46	3.92	< 0.001
ii	DHEA (ng/g)	Intercept	1399.99	38.75	36.13	< 0.001
		Month	-17.08	6.22	-2.75	0.006
iii	Aldosterone (ng/g)	Intercept	40.71	3.38	12.03	< 0.001
		Month	1.39	0.54	2.57	0.010
**Species: *Tursiops aduncus***						
iv	Cortisol (ng/g)	Intercept	194.99	33.58	5.81	< 0.001
		Month	19.28	5.77	3.34	0.001
v	Cort:DHEA Ratio	Intercept	0.13	0.02	5.79	< 0.001
		Month	0.02	0.00	4.24	< 0.001
vi	Aldosterone (ng/g)	Intercept	133.98	8.39	15.98	< 0.001
		Sex	-22.48	6.60	-3.41	0.001
		Month	-11.45	1.30	-8.79	< 0.001
vii	DHEA (ng/g)	Intercept	1513.89	103.73	14.59	< 0.001
		Sex	-226.03	71.30	-3.17	0.002
		Age	12.93	3.40	3.81	< 0.001
		Month	-38.04	13.56	-2.81	0.006
**Species: *Delphinapterus leucas***					
Viii	Cort:DHEA Ratio	Intercept	0.95	0.014	6.77	< 0.001
		Age	0.003	0.001	3.66	0.001

**Table 5:** General reference intervals or values for fecal hormone values for healthy cetaceans. General reference intervals or values (without accounting for sex, age, age^2^, and month) are given below if access to the iOS ZooPhysioTrak app is not available for a facility.

Variable	n	Reference Interval / Values
**Species: *Tursiops truncatus***		
Cortisol (ng/g)	1338	0.00–354.04
DHEA (ng/g)	1331	413.44–2010.31
Aldosterone (ng/g)	1370	0.00–113.46
Cort:DHEA Ratio	1333	0.00–0.30
**Species: *Tursiops aduncus***		
Cortisol (ng/g)	224	54.37–468.35
DHEA (ng/g)	230	537.48–2309.20
Aldosterone (ng/g)	219	0.00–128.83
Cort:DHEA Ratio	221	0.03–0.35
**Species: *Delphinapterus leucas***		
Cortisol (ng/g)	74	44.80–252.76
DHEA (ng/g)	78	548.06–1807.08
Aldosterone (ng/g)	80	0.00–139.32
Cort:DHEA Ratio	73	0.04–0.22
**Species: *Lagenorhynchus obliquidens***		
Cortisol (ng/g)	43	63.97–234.22
DHEA (ng/g)	46	549.76–1643.75
Aldosterone (ng/g)	43	0.00–80.50
Cort:DHEA Ratio	44	0.05–0.23

*Note*. n is the total number of samples.

## Discussion

Historically, cortisol and aldosterone have been the most common indicators used for bottlenose dolphins and beluga whales [32, 33]. The current study builds on the existing literature by providing reference intervals or values for two common indicators of welfare (*i*.*e*., cortisol and aldosterone) for four species of cetaceans. While more information is clearly needed, reference intervals or values are also provided for one potential novel (*i*.*e*., cortisol:DHEA) indicator of welfare. DHEA, or more specifically the ratio of fecal cortisol metabolites to fecal DHEA metabolites provides a novel potential indicator for comparison across individuals. Facilities can use the ZooPhysioTrak application to determine how individual animal’s values compare to healthy individuals in this project. Facilities without an iOS device can use the less accurate but still important reference intervals or values provided in this manuscript. Overall, this information can help facilities make informed management decisions and allow them to monitor the animals to ensure each individual falls within healthy ranges. It is important to note that when utilizing the less accurate reference intervals or values presented in the manuscript, these do not account for sex, age, age^2^, and month. Additionally, these intervals or values do not include effect of facility and this should be considered when interpreting values and making management or veterinary decisions.

While the r^2^ values in the current study are quite low, explaining very little variance in the values, the goal of the current research was not to explain all sources that contribute to the three analytes. The objective was to create a useful tool that can be utilized by animal care professionals that work with these species by presenting the range of values observed in healthy individuals. In addition, the r^2^ values observed during the current study are similar to values in previous reports of reference intervals for cortisol and aldosterone for wild dolphins [16]. Additionally, it is important to note that the parallelism was not significant and the inter-assay CV was quite high for the aldosterone assay. While samples were rerun when intra-assay CV was greater than 15%, the values should still be interpreted with caution. Future research should examine in more detail why aldosterone assays can be challenging for dolphins compared to other markers of adrenal activity [7, 16].

While age was a significant predictor of cortisol for *Tursiops truncatus* in the current study, age has not been a significant predictor in previous reports [16, 34]. Month was a significant predictor of cortisol for *Tursiops aduncus* in the current study, similar to seasonal variations observed in previous work [31]. Overall, it would appear that for the species in the current study, environmental, social, and management factors likely have a larger impact on adrenal activity [3, 35–37]. There are indeed many environmental influences that impact adrenal activity in cetaceans that were not examined in the current study. Finally, due to differences in assays, sample type, and laboratories, direct comparisons of the actual values between studies are not possible.

While there have been many informative studies examining adrenal activity in cetaceans [7, 16], the majority have been based on blood values. The current study offers an alternative less invasive method to monitor four species of cetaceans using fecal samples from a single point or over time. While the sample sizes for some of the species are on the low end, these data still provide a starting point to monitor individual animals. The sample size for common bottlenose dolphins provides some robust estimates for both common and one potential novel indicator of animal welfare. Moving forward it is recommended that facilities use identical methods when making comparisons to these reference intervals or values as other methods or assay supplies could provide different results. It is also recommended in the future that more facilities with those species assess these same indicators to contribute to the literature and continue to build a larger sample size. In addition, while there were four species involved in the current study, additional species could benefit from having reference intervals or values for these fecal metabolite hormones.

As previously noted, the study design incorporated a number of health factors commonly used in facilities to gauge health status before animals were included in the analysis. However, we cannot guarantee that animals were not experiencing some level of stress, and should be kept in mind when interpreting results to make management decisions. As in any evaluation of health, veterinarians also reply on monitoring for indicators of normal behavior and cohort relationships from direct observation as well as information from animal care staff involved in the animal’s daily lives. Moving forward, facilities can now monitor four species of cetaceans using the provided methods and reference intervals or values depending on the species. However, it is important to note that inter-laboratory variation could present a problem as bias can exist when examining adrenal activity across different types of biological samples [38, 39]. The solution would be to utilize the same laboratory to ensure that results are valid to make informed management decisions. Alternatively, duplicate samples could be analyzed by additional laboratories in conjunction with the current laboratory to convert new reference intervals or values for comparison using appropriate statistical techniques [40]. Future research should also examine how environmental, seasonal, and animal management characteristics (*e*.*g*., social structure, animal training, etc.) influence adrenal activity in these species. That information could also help guide animal management decisions to help ensure high levels of animal welfare.

Although novel, the ratio of cortisol:DHEA fecal metabolites could be an indicator to help ensure individual animals are thriving and elevated levels could suggest that intervention or management changes are necessary. The fact that animals can be easily trained for sample collection and is relatively less-invasive makes the method even more beneficial for monitoring the welfare of the animals. The creation of ZooPhysioTrak also puts information into the hands of zoological professionals that can use the results to make informed decisions. Zoological institutions continuously strive to provide optimum physical and mental health for animals throughout their lifetime. Development and incorporation of new health tools and information necessary for application is critical for the animals under their professional care to maintain and elevate welfare for individuals.

## Supporting information

S1 Statistics (XLSX)
